# Lymphatic Vasculature in Energy Homeostasis and Obesity

**DOI:** 10.3389/fphys.2020.00003

**Published:** 2020-01-22

**Authors:** Yen-Chun Ho, R. Sathish Srinivasan

**Affiliations:** ^1^Cardiovascular Biology Research Program, Oklahoma Medical Research Foundation, Oklahoma City, OK, United States; ^2^Department of Cell Biology, University of Oklahoma Health Sciences Center, Oklahoma City, OK, United States

**Keywords:** *Prox1*, vegfc, neuropilin, lymphedema, lipedema, obesity, inflammation

## Abstract

Obesity is a leading cause of cardiovascular diseases and cancer. Body mass is regulated by the balance between energy uptake and energy expenditure. The etiology of obesity is determined by multiple factors including genetics, nutrient absorption, and inflammation. Lymphatic vasculature is starting to be appreciated as a critical modulator of metabolism and obesity. The primary function of lymphatic vasculature is to maintain interstitial fluid homeostasis. Lymphatic vessels absorb fluids that extravasate from blood vessels and return them to blood circulation. In addition, lymphatic vessels absorb digested lipids from the intestine and regulate inflammation. Hence, lymphatic vessels could be an exciting target for treating obesity. In this article, we will review our current understanding regarding the relationship between lymphatic vasculature and obesity, and highlight some open questions.

## Introduction

**FIGURE 1:**
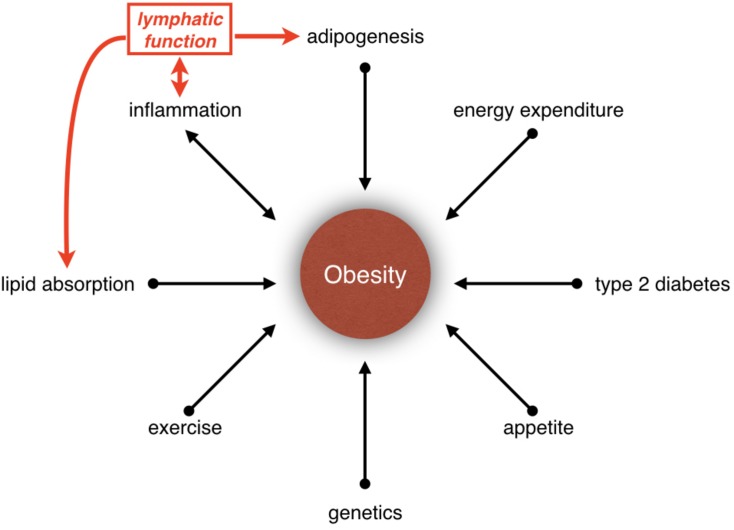
Relationship between obesity and lymphatic vasculature. Obesity is influenced by many parameters, such as inflammation, lipid absorption, adipogenesis, type 2 diabetes, appetite, genetics, exercise, and energy expenditure. Increasing evidences have suggested that the lymphatic vasculature could be participating in the development of obesity by regulating lipid absorption, adipogenesis and inflammation.

Obesity is a disease caused by energy imbalance (Hill et al., 2012; Tchernof and Despres, 2013). Appetite, food consumption and exercise are only some of the parameters that regulate energy balance. In fact, obesity is a polygenic disease, and its etiology is determined by multiple factors such as neural circuits, hormones, nutrient absorption, lipid storage, lipid metabolism and inflammation (Figure 1). Besides, obesity is a risk factor for cancer and cardiovascular diseases such as type 2 diabetes mellitus, hypertension, hypercholesterolemia, hyperlipidemia, coronary artery disease, and stroke. In the United States, it is estimated that one-third of adults and one-sixth of children are obese (Ogden et al., 2013; Cunningham et al., 2014). The economic impact of obesity is estimated to be more than $100 billion per year in the United States alone (Hammond and Levine, 2010). Hence, better understanding of the mechanisms that regulate obesity and approaches to treat this disease are urgently needed. In this article, we will describe exciting new discoveries regarding the role of lymphatic vasculature in metabolic regulation and obesity.

## Functions of the Lymphatic Vasculature

Lymphatic vasculature absorbs interstitial fluid and returns it to blood circulation (Tammela and Alitalo, 2010; Geng et al., 2017). Lacteals are specialized lymphatic vessels in the intestine, which absorb digested lipids (Bernier-Latmani and Petrova, 2017; Petrova and Koh, 2018). Lymphatic vessels are also important for clearing low-density lipoprotein particles from the skin and arteries by reverse cholesterol transport (Martel et al., 2013). Furthermore, lymphatic vasculature regulates the immune response (Randolph et al., 2005). Lymphatic endothelial cells (LECs) secrete cytokines such as CCL19 and CCL21 to recruit activated dendritic cells and transport them to lymph nodes to promote T- and B-cell activation (Randolph et al., 2005). LECs could directly present antigens to immune cells and promote peripheral immune tolerance (Rouhani et al., 2014). In summary, lymphatic vasculature regulates fluid homeostasis, lipid absorption, and immune response.

## Lymphatic Vascular Architecture

Lymphatic vasculature is made of LECs. LECs express unique markers that distinguish them from blood vascular endothelial cells. These markers include prospero-related homeobox 1 transcription factor (PROX1) (Wigle and Oliver, 1999), the tyrosine kinase receptor vascular endothelial growth factor 3 (VEGFR3/Flt4) (Kaipainen et al., 1995), the transmembrane *O*-glycoprotein podoplanin (Breiteneder-Geleff et al., 1999) and neuropilin 2 (NRP2) (Yuan et al., 2002). Endothelial cell junction proteins such as VE-cadherin, claudin-5, and PECAM-1 are expressed in all LECs. However, these junctional molecules are organized in different patterns to generate two functionally distinct structures within the lymphatic vasculature: lymphatic capillaries and collecting lymphatic vessels. Lymphatic capillaries have discontinuous “button-like junctions,” which allows immune cells, interstitial fluid, and digested lipids to enter the vessels (Baluk et al., 2007; Yao et al., 2012). Lymphatic capillaries are connected to the surrounding tissues by anchoring filaments, which respond to changes in external fluid pressure by opening the button-like junctions to allow the entry of leukocytes and interstitial fluid into the vessels (Ikomi et al., 1996; Triacca et al., 2017). Unlike lymphatic capillaries, collecting vessels have continuous “zipper-like” endothelial junctions that prevent lymph leakage (Baluk et al., 2007; Yao et al., 2012). Collecting vessels are covered with continuous basement membrane and lymphatic muscle cells with contractile ability (Bridenbaugh et al., 2003; von der Weid and Zawieja, 2004; von der Weid, 2019). Collecting lymphatic vessels contain lymphatic valves (LVs), which regulate the unidirectional flow of lymph. Lymph flows through a series of lymph nodes where the immune cells and antigens are filtered out. Ultimately lymph is returned to blood circulation through two-pairs of lymphovenous valves (LVVs) located bilaterally at the junction of jugular and subclavian veins (Oliver and Srinivasan, 2008; Geng et al., 2017).

In the intestine, cholesterol, long-chain fatty acids, and fat-soluble vitamins are absorbed by intestinal epithelial cells, and repackaged in enterocytes into large (200–1000 nm) triglyceride-loaded particles called “chylomicrons” (Tso and Balint, 1986; Zhang et al., 2018). Each intestinal villus contains a single lacteal, which is a lymphatic capillary with button-like junctions (Zhang et al., 2018). Most chylomicrons are absorbed by the lacteals, from which they are transported to submucosal and mesenteric collecting lymphatic vessels, then into the thoracic duct, and finally to the blood circulation (Oliver and Srinivasan, 2008; Randolph and Miller, 2014). Thus, digested lipids bypass the liver and are accessible to other organs and cells prior to the liver (Ahn and Park, 2016).

## Lymphatic Vascular Dysfunction Results in Lymphedema

Structural and functional defects of lymphatic vascular system can lead to lymphedema, which is characterized by localized accumulation of interstitial fluid and tissue swelling (Lohrmann et al., 2009). Although lymphedema is not a life-threatening disease, it could cause functional disability, skin infection, inflammation fibrosis and pain (Mehrara and Greene, 2014; Escobedo and Oliver, 2016; Breslin et al., 2018). Lymphedema is classified into either primary or secondary lymphedema. Primary lymphedema is caused by mutations in genes that regulate lymphatic vascular development. These genetic defects result in structural and functional defects in LECs, LVs, or LVVs. Primary lymphedema is typically present early in life (infancy, childhood, or puberty), and rarely appears in adulthood (older than age of 35) (Grada and Phillips, 2017). Secondary lymphedema is caused by surgery (removal of lymph nodes) or radiation therapy in cancer patients, infection or trauma. Secondary lymphedema is much more prevalent than primary lymphedema, and is an increasing clinical problem due to the improving survival rates of cancer patients (Whelan et al., 2015; Rockson et al., 2019).

## Clinical Correlations Between Obesity And Lymphedema

There is reciprocal relationship between obesity and lymphatic dysfunction. Obesity is one of the risk factors for the development of secondary lymphedema in breast cancer patients (Werner et al., 1991; Helyer et al., 2010; Mehrara and Greene, 2014). In a study that compared 137 breast cancer patients, those with a body mass index (BMI) greater than 30 showed three times higher risk for developing upper extremity lymphedema compared with patients with lower BMI (Helyer et al., 2010). A similar result was observed in a second independent study involving 282 breast cancer patients (Werner et al., 1991).

Obesity could result in lymphedema in the absence of other risk factors such as primary lymphedema, inguinal lymphadenectomy or radiation treatment (Greene et al., 2012; Arngrim et al., 2013). In a study with a small cohort of 15 obese individuals, the average BMI of the patients with lymphedema (70.1) was much higher than the BMI of obese patients without lymphedema (42.0) (Greene et al., 2012). Another clinical study demonstrated that lean, healthy men (BMI: 22.3) showed better adipose tissue lymphatic drainage when compared with obese, but otherwise healthy men with normal glucose tolerance (BMI: around 35.7) (Arngrim et al., 2013).

Impaired lymphatic function could also lead to adipose tissue accumulation and fibrosis (Brorson, 2003). Secondary lymphedema patients tend to have higher BMI compared to breast cancer survivors without lymphedema (Ahmed et al., 2008). Lymphedema patients have more adipocytes in the edematous tissues. The edematous tissues are infiltrated with macrophages and lymphocytes, which promote the proliferation and hypertrophy of local adipocytes (Brorson, 2012; Ghanta et al., 2015). Adipose tissue-derived stem cells from lymphedema patients are more potent in their ability to undergo adipogenic differentiation *in vitro* when compared to cells derived from control patients (Levi et al., 2013). Taken together, these results suggest that chronic lymphedema creates an inflamed environment, which promotes adipose tissue accumulation (Cucchi et al., 2017). Nevertheless, lymphedema could reduce mobility and thereby reduce energy expenditure. Hence, is adipogenesis a direct outcome of lymphedema or is it indirectly caused by chronic immobility that is associated with lymphedema? This important question was addressed in part by recent studies, which have suggested that exercise (yoga, stretching, strength training) could improve breast cancer-related lymphedema (Baumann et al., 2018; Panchik et al., 2019). These studies must be very encouraging to lymphedema patients as they suggest that lymphedema-associated adipogenesis could be controlled by exercise.

## Lipedema: A Painful Disease That Affects Lymphatic Vasculature and Adipocytes

Lipedema is a disease characterized by the swelling of the legs due to the deposition of subcutaneous adipose tissue in the legs, thighs and buttocks (Torre et al., 2018). Most lipedema patients have high BMI, either in the overweight (25 to 30) or obese range (>30) (Fife et al., 2010). However, lipedema patients are less prone to type 2 diabetes mellitus, hypertension and dyslipidemia indicating that lipedema is distinct from obesity (Fonder et al., 2007; Al-Ghadban et al., 2019). Wold et al. proposed a series of criteria to diagnose lipedema in 1940s that is still in use (Child et al., 2010). These criteria include: (1) occurrence almost exclusively in women; (2) bilateral and symmetrical nature of adipose tissue accumulation with minimal involvement of the feet or ankle; (3) minimal pitting edema; (4) pain, tenderness and easy bruising; (5) persistent swelling of lower extremities despite elevation or weight loss (Child et al., 2010). The cause of lipedema is still unclear; however, there is evidence that hormonal and hereditary influence may play a role in the development of lipedema (Fonder et al., 2007). Important for this work, lipedema patients often show abnormal lymphoscintigraphic pattern with slow lymph flow (Bilancini et al., 1995). Ultrasound, MRI, and lymphangiogram are helpful for clinical diagnosis of lipedema (Warren Peled and Kappos, 2016). However, the test results may show “normal lymphatic function” in the early stage of the disorder (Fonder et al., 2007; Lohrmann et al., 2009; Child et al., 2010). Consequently, lipedema is often misdiagnosed as obesity (Fonder et al., 2007; Lohrmann et al., 2009; Godoy Mde et al., 2012; Shavit et al., 2018). Hence, whether lymphatic stasis in these patients cause increased adipogenesis or vice versa remains unknown.

## Mouse Models That Highlight the Role of Lymphatic Vasculature on Metabolism and Obesity

As described in the previous sections, there is strong clinical correlation between lymphatic dysfunction and adipogenesis. In this section we will discuss evidence from mouse models, which allows us to determine causality.

### PROX1

*Prox1* heterozygous mice develop chylous ascites (leakage of lipid−rich lymph into the peritoneal cavity) soon after birth (Harvey et al., 2005). These *Prox1*^+/−^ mice develop adipocyte hypertrophy (increase in the size of adipocytes), increased serum free fatty acids, fatty liver and obesity in adulthood. Conditional deletion of one allele of *Prox1* from endothelial cells recapitulated this phenotype. In addition, overexpression of *Prox1* in the LECs rescues the obese phenotype of *Prox1* heterozygous mice (Escobedo et al., 2016). Furthermore, lymph could stimulate adipogenesis *in vitro* (Harvey et al., 2005). Together these results suggest that leakage of lymph triggers the onset of adipogenesis and obesity in *Prox1*^+/−^ mice (Harvey et al., 2005; Escobedo et al., 2016).

Obesity is mainly defined as a disease of energy imbalance. Young non-obese *Prox1*^+/−^ mice do not consume more food compared to their control littermates. In contrast, older obese *Prox1*^+/−^ mice consume less food and exercise less when compared with controls (Harvey et al., 2005). What promotes the transition between these metabolic states is currently unknown. Adipocyte inflammation is known to reduce energy expenditure and lower food consumption (Leibel et al., 1995; Zhao et al., 2018). Hence, it is possible that the adipocyte inflammation observed in *Prox1*^+/−^ mice (Harvey et al., 2005) might be contributing to the onset of obesity in older mice.

The obese phenotype of *Prox1*^+/−^ mice is strain dependent. *Prox1*^+/−^ and Tie2-Cre; *Prox1*^+/f^ mice bred in the NMRI background develop obesity (Harvey et al., 2005). However, conditional deletion of one allele of *Prox1* using lymphatic vasculature-specific *Lyve1-Cre* in a non-NMRI background does not result in obesity (Escobedo et al., 2016). The reason for this strain-specific onset of obesity remains to be determined. Nevertheless, PROX1 is expressed in many metabolism-related organs (see below). Hence, it is worth investigating whether PROX1 might be playing additional LEC-independent roles in regulating obesity.

PROX1 is necessary for the development of liver (Sosa-Pineda et al., 2000; Seth et al., 2014). Obese *Prox1*^+/−^ mice develop hepatosteatosis (fatty liver disease) (Harvey et al., 2005). This observation is consistent with the fact that PROX1 is important for the maintenance of lipid homeostasis in the liver. In the hepatocytes PROX1 interacts with HDAC3 to down-regulate the expression of genes such as *G0s2*, *Elovl6*, *Mfsd2a*, and *Cidec*, which are involved in lipid synthesis and lipolysis (Charest-Marcotte et al., 2010; Armour et al., 2017). Reducing PROX1 expression by AAV-based shRNA resulted in an increase in triglyceride levels in the mouse liver (Armour et al., 2017). In addition, treatment of mice with rapamycin causes hyperlipidemia by lowering PROX1 expression in the liver (Kwon et al., 2016).

PROX1 is important for the development of pancreatic endocrine cells and glucose metabolism. Conditional deletion of *Prox1* in pancreatic progenitors of mice showed delayed embryonic islet cell genesis and damage of ductal tissue in adulthood (Westmoreland et al., 2012). Overexpression of *Prox1* in immature β-cells promotes acute hyperglycemia (Paul et al., 2016). Consistent with these reports single-nucleotide polymorphisms in *PROX1* are associated with higher fasting glucose levels and type 2 diabetes mellitus (Lecompte et al., 2013; Kretowski et al., 2015; Hamet et al., 2017; Adamska-Patruno et al., 2019). Furthermore, hyperinsulinemia is observed in obese *Prox1*^+/−^ mice (Harvey et al., 2005). However, whether it is due to lymphatic dysfunction or pancreatic defects is currently unknown. Insulin treatment is associated with weight gain in human patients (Russell-Jones and Khan, 2007). Hence, whether increased plasma insulin levels might contribute to obesity in *Prox1*^+/−^ mice could be investigated.

Skeletal muscle is an important tissue for glucose metabolism and fat (mainly free fatty acid) storage (Frontera and Ochala, 2015). Besides, metabolic rate of skeletal muscles is an important index of resting energy expenditure (Zurlo et al., 1990). Skeletal muscles are composed of slow muscle fibers and fast muscle fibers, which generate energy through aerobic and anaerobic mechanisms respectively. Obesity in humans is inversely correlated with proportion of slow muscle fibers (Wade et al., 1990). *Prox1* is expressed in the satellite cells and slow muscle fibers. Conditional deletion of *Prox1* in skeletal muscles switched the slow muscle fibers to fast muscle fibers (Kivela et al., 2016). In contrast, overexpression of PROX1 converted fast muscle fibers to slow muscle fibers. These results suggest that *Prox1* may play a critical metabolic role by regulating the identity of muscle fibers (Kivela et al., 2016).

*Prox1* is expressed in brain regions, such as cortex, hippocampus, thalamus, hypothalamus, and cerebellum during embryonic and post-natal stages (Galeeva et al., 2007; Lavado and Oliver, 2007). Importantly, PROX1 is expressed in the paraventricular nucleus and the arcuate nucleus of the hypothalamus (Lavado and Oliver, 2007), which are directly involved in appetite control and feeding behavior (Formolo et al., 2019). *Prox1* is a key factor for granule cell formation, maturation and differentiation during developmental stages (Lavado et al., 2010). *Prox1* is important for adult neurogenesis in dentate gyrus and subventricular zone (Lavado et al., 2010; Bunk et al., 2016). However, whether PROX1 regulates the formation and/or functioning of the appetite centers of the brain remains to be investigated.

PROX1 is not expressed in retroperitoneal adipocytes (Harvey et al., 2005). However, PROX1 is reported to be expressed in subcutaneous and omental adipose tissues (Procino, 2014). Whether PROX1 is expressed in adipocyte progenitors and whether it could regulate adipocyte lineage in an epigenetic manner is unknown.

In summary, based on the known roles of PROX1 in metabolic tissues we are tempted to speculate that PROX1 might be controlling certain aspects of obesity in non-lymphatic tissues. Obesity has not been reported in mice that are heterozygous for *Prox1* specifically in the liver, pancreas, brain, or muscle. Hence, it is possible that obesity arises in *Prox1*^+/−^ mice as a consequence of metabolic defects in multiple tissues as reported for another transcription regulator TRIM28 (Dalgaard et al., 2016). PROX1 regulates the expression of mitochondrial lipid transporter CPT1a in LECs (Wong et al., 2017). Through this metabolic pathway PROX1 promotes epigenetic changes that support lymphangiogenesis. Whether PROX1 regulates CPT1a expression in other tissues, and whether this pathway could contribute to the onset of obesity in *Prox1*^+/−^ mice needs to be evaluated.

### VEGF-C, VEGF-D, and VEGFR3

VEGF-C/VEGFR3 signaling is critical for lymphatic vascular development. *Vegfc^–/–^* mice lack lymphatic vessels due to a failure of LEC budding from the embryonic veins (Karkkainen et al., 2004; Hagerling et al., 2013). Mutations in *VEGFR3* and *VEGF-C* are associated with congenital lymphedema (Ferrell et al., 1998; Brice et al., 2005; Gordon et al., 2013). Subcutaneous fat is observed in *Chy* mice carrying an inactivating point mutation in the kinase domain of VEGFR3 (Karkkainen et al., 2001). However, obesity is not observed in *Chy* mice (Aspelund et al., 2016) indicating that obesity and adipogenesis are not necessarily correlated.

Deletion of *Vegfc* in adult mice caused the regression of intestinal lacteals, reduced lipid absorption and provided resistance to high fat diet-induced obesity (Nurmi et al., 2015). Likewise, K14-VEGFR3-Ig mice in which a soluble form of extracellular VEGFR3 (sVEGFR3) that traps VEGF-C and VEGF-D was expressed from keratinocytes, were also resistant to high fat diet induced obesity, hepatic lipid accumulation and metabolic dysfunction (Karaman et al., 2015). In contrast, overexpression of VEGF-C in the keratinocytes resulted in weight gain, hepatic lipid accumulation, subcutaneous adipose tissue accumulation and insulin resistance even in chow diet fed mice (Karaman et al., 2016). Furthermore, an increased number of pro-inflammatory macrophages were found in the white adipose tissue of the VEGF-C overexpression mice (Karaman et al., 2016). These results suggest that the pro-lymphangiogenic VEGF-C is also a pro-inflammatory and pro-obesogenic molecule. These results are consistent with the observation that obese patients have elevated levels of VEGF-C in their serum (Silha et al., 2005; Wada et al., 2011).

Gut microbiota are important regulators of metabolism and energy balance (Nicholson et al., 2012; Chevalier et al., 2015). Depletion of gut microbiota leads to morphological defects in lacteals (Suh et al., 2019). Gut microbiota stimulates macrophages of the intestinal villi to produce VEGF-C. Lack of gut microbiota causes the transformation of button-like junctions of lacteals into zipper-like junctions due to reduced VEGF-C signaling (Suh et al., 2019). This junctional transformation reduced lipid absorption in the gut (Suh et al., 2019). Taken together, these results suggest that gut microbiota may play a role in obesity and metabolic syndrome by regulating VEGF-C signaling.

VEGF-D is an additional ligand of VEGFR3. Deletion of *Vegfd* in mice does not result in any obvious lymphatic defects (Baldwin et al., 2005). However, overexpression of VEGF-D can induce lymphangiogenesis and angiogenesis under normal and pathological conditions (Baldwin et al., 2005; Bui et al., 2016). Overexpression of VEGF-D in lungs, kidneys, and adipose tissue induces hyperplasia of lymphatic vessels and lymphangiectasia (Lammoglia et al., 2016). Moreover, long-term overexpression of VEGF-D in adipose tissue of mice caused increased macrophage infiltration and enhanced adipose tissue fibrosis (Lammoglia et al., 2016). However, overexpression VEGF-D did not result in obesity or insulin resistance in chow diet fed mice (Lammoglia et al., 2016). Surprisingly, in high-fat diet fed condition overexpression of VEGF-D in the adipocytes resulted in enhanced glucose clearance, lower insulin levels and reduced liver triglycerides (Chakraborty et al., 2019). Total F4/80^+^ macrophages were reduced in subcutaneous adipose tissue by increased immune trafficking from the tissue. These results suggest that enhanced VEGF-D signaling in the adipose tissue might reduce obesity associated-immune cell accumulation and improve metabolic response (Chakraborty et al., 2019).

These unexpected and somewhat contradictory roles of VEGF-C, VEGF-D, and VEGFR3 call for further investigation of this signaling pathway in metabolic disorders and obesity.

### Neuropilin

Neuropilins 1 and 2 (NRP1 and NRP2) were originally identified as molecules that are necessary for the patterning of neurons (Kitsukawa et al., 1995; Kolodkin et al., 1997). NRPs were found to function as co-receptors of VEGF-receptors in endothelial cells (Bouvree et al., 2012). NRP1 is mainly expressed in arteries and NRP2 is expressed in veins and lymphatic vessels (Herzog et al., 2001; Yuan et al., 2002). VEGF-A associates with NRP1/VEGFR1 complex to induce angiogenesis (Soker et al., 1998; Zhang et al., 2018). VEGF-C associates with NRP2/VEGFR2/VEGFR3 complex to regulate lymphangiogenesis (Favier et al., 2006; Bouvree et al., 2012; Deng et al., 2015). NRPs also interact with secreted and transmembrane ligands known as semaphorins (Kolodkin et al., 1997). There are eight classes of SEMA family (SEMA1-7; SEMAV); of which, SEMA3-7 are found in vertebrates (Neufeld and Kessler, 2008). NRPs can interact with members of SEMA3 family. SEMA3/NRP complexes further interact with transmembrane proteins called plexins. Plexins regulate the development of many organs, such as skeleton and kidney, and participate in angiogenesis and vascular patterning (Perala et al., 2012). SEMA3/NRP1/PlexinA1 complex regulates LV morphogenesis (Bouvree et al., 2012; Jurisic et al., 2012).

*Nrp1* knockout mice are embryonic lethal at E12.5. *Nrp1^–/–^* mice exhibited vascular defects such as agenesis and transposition of great vessels, and disorganized and insufficient development of vascular networks in the yolk sac (Kawasaki et al., 1999). Global overexpression of *Nrp1* results in ectopic blood vascular sprouting, dilated vessels, and abnormal heart (Kitsukawa et al., 1995).

Lacteals are made of LECs with discontinuous button-like junctions. These button-like junctions allow the easy passage of large chylomicrons into the lacteals. Deletion of *Nrp1* or *Vegfr1* by inducible endothelial- specific Cre resulted in the transition of button-like junctions of lacteals into zipper-like junctions (Zhang et al., 2018). Knock out of *Nrp1* or *Vegfr1* in blood endothelial cells enhances the bioavailability of VEGF-A and downstream signaling through VEGFR2 in LECs. High VEGFR2 signaling activity promotes zipper-like junctions in lacteals. Increased number of zipper-like junctions in mice lacking NRP1 or VEGFR1 reduced chylomicron uptake and also increased their resistance to high-fat diet-induced obesity (Zhang et al., 2018).

*Nrp2*-null mice are viable but have hypoplastic lymphatic capillaries compared with littermate controls (Yuan et al., 2002). Whether LEC-specific deletion of *Nrp2* affects metabolism is currently not known.

Neuropilins and their ligands play an additional-complex role in energy homeostasis through the nervous system (van der Klaauw et al., 2019). Several variants of *NRP1*, *NRP2*, and *SEMA3* are identified in severely obese individuals (van der Klaauw et al., 2019). These variants disrupt the secretion of melanocortin and/or signaling downstream of melanocortin-4-receptor (MC4R) in human embryonic kidney 293 cells. *In vivo* melanocortin is released by pro-opiomelanocortin (POMC) or Neuropeptide Y (NPY)/Agouti-related protein (AgRP) positive neurons in hypothalamus. Melanocortin/MC4R signaling reduces food intake and increase energy expenditure (Cowley et al., 2001). Deletion of *Nrp2* in POMC neurons reduces energy expenditure and causes weight gain (van der Klaauw et al., 2019). Hence, the lymphatic and non-lymphatic contribution of NRPs should also be dissected in the context of obesity.

### Apelin

Apelin is a peptide and a ligand for the orphan G protein-coupled receptor AJP (Tatemoto et al., 1998). Apelin and AJP are widely expressed in the brain, intestine, kidney, adipose tissue and vasculature (Lee et al., 2000; Chen et al., 2003). Apelin/AJP signaling is involved in many physiological processes, including glucose homeostasis, lipid metabolism (Trayhurn et al., 2006), obesity (Boucher et al., 2005; Sawane et al., 2013), and diabetes (Li et al., 2006). High fat diet-fed *Apln^–/–^* mice were severely obese and had lymphatic and blood vasculature abnormalities (Sawane et al., 2013).

*In vitro* studies determined that Apelin could stabilize the expression of adherens junction protein VE-Cadherin and reduce fatty acid-induced vascular hyperpermeability (Sawane et al., 2013). These results suggest that Apelin maintains lymphatic vessel integrity, and inhibits dietary fat absorption and accumulation.

In summary, these observations suggest a context dependent role of lymphatic vasculature in regulating obesity. Nevertheless, additional non-LEC roles for these molecules in regulating metabolism cannot be excluded.

## Mouse Models of Obesity With Defective Lymphatic Function

As mentioned previously, lymphatic function is abnormal in obese humans (Arngrim et al., 2013). The following mouse studies support those observations and provide mechanistic explanations for obesity-induced lymphatic dysfunction.

### Adipokines

Adipocytes secrete signaling molecules such as leptin, adiponectin, and resistin. These signaling molecules are collectively known as adipokines. One of the primary functions of leptin is to activate nerve centers of the brain and promote satiety. *Ob/Ob* mice, which lack leptin develop severe obesity and type 2 diabetes mellitus. Scallan et al. (2015) demonstrated that the lymphatic vessels of *Ob/Ob* mice are dilated and leaky. They further showed that this lymphatic phenotype is due to low nitric oxide (NO) bioavailability (Scallan et al., 2015).

Adiponectin also plays a role in maintaining lymphatic function (Shimizu et al., 2013). Using tail-injury model Shimizu et al. demonstrated that mice lacking adiponectin were deficient in their ability to clear tissue fluid and had severe tail edema. In contrast, administration of adiponectin promoted the regrowth of lymphatic vessels and reduced edema. Mechanistically, adiponectin activated the phosphorylation of AKT and eNOS through AMPK and promoted the survival and proliferation of LECs. These data suggest that adipokines such as leptin and adiponectin act as modulators of lymphatic function.

### High-Fat Diet-Induced Obesity

Several studies have investigated the influence of high fat diet induced obesity on lymphatic vascular function (Lim et al., 2009; Blum et al., 2014; Savetsky et al., 2014, 2015; Barton and Husmann, 2016; Garcia Nores et al., 2016; Hespe et al., 2016; Nitti et al., 2016; Torrisi et al., 2016). They overwhelmingly conclude that obesity affects lymphatic vascular formation and function.

High-fat diet aggravates hypercholesterolemia in *ApoE^–/–^* mice. Lymphatic vessels of these hypercholesterolemic mice were defective in their ability to take up interstitial fluid and were leaky (Lim et al., 2009). Moreover, collecting lymphatic vessels of these mice lacked smooth muscle cell coverage and had abnormal LVs (Lim et al., 2009). Therefore, hypercholesterolemia could inhibit the maturation of lymphatic vessels.

Weitman et al. (2013) demonstrated that high-fat diet-induced obesity resulted in impaired lymphatic fluid uptake and transport, reduced number of LECs in lymph nodes, dysregulated CCL21 gradient and defective immune cell trafficking. Similarly, Detmar and colleagues showed that high-fat diet impaired the contractility of lymphatic vessels and made them non-responsive to mechanostimulation (Blum et al., 2014). Thus, high-fat diet could inhibit LEC survival or proliferation and could compromise lymphatic vascular function.

How could obesity affect lymphatic function? Using tail-injury model Detmar and colleagues quantified lymphatic function in high-fat diet-fed mice before they became obese (Gousopoulos et al., 2017). They determined that lymph transport is identical between control and high-fat diet-fed mice. This important observation indicated that increased number or size of adipocytes, but not serum lipids, is responsible for lymphatic dysfunction in obese mice. Obesity is associated with chronic, low-grade inflammation (Wellen and Hotamisligil, 2005). Savetsky et al. (2014, 2015) demonstrated that obesity-induced inflammation could compromise lymphatic function. In contrast, inhibition of inflammation increases lymphatic capillary density and restores lymphatic vascular function in high-fat diet-induced obese mice (Torrisi et al., 2016). Taken together, inflammation could play a key role in obesity-induced lymphatic dysfunction. Furthermore, lymphatic dysfunction could also exacerbate inflammation, thus setting up a vicious feedback loop (Figure 1). Compression or increased lymph load caused by adipocyte hypertrophy/hyperplasia could also affect lymphatic function.

## Summary and Future Directions

Obesity is a complex disease that is influenced by many parameters (Figure 1). Evidence is clear that the lymphatic vasculature regulates lipid absorption, adipogenesis, and inflammation (Figure 1). It is also clear that obesity could affect lymphatic function. However, whether lymphatic dysfunction could cause obesity is not fully clear. Mouse studies have revealed that while some mutations that cause lymphatic vascular defects could promote obesity, other mutations protect against it. As proteins generally perform multiple functions it is possible that the genes that regulate lymphatic vascular development or functioning could independently regulate metabolism. Additionally, lymphedema causes severe pain and immobility in patients, which in turn could affect energy expenditure and cause weight gain. Due to these complexities carefully controlled clinical studies and mouse experiments are needed to better understand the relationship between lymphatic function and metabolic diseases. This knowledge could significantly impact the lives of millions of lymphedema, lipedema, and obesity patients worldwide.

## Author Contributions

Y-CH and RS designed, co-wrote, and coedited the manuscript.

## Conflict of Interest

The authors declare that the research was conducted in the absence of any commercial or financial relationships that could be construed as a potential conflict of interest.
